# CDK-associated Cullin 1 can promote cell proliferation and inhibit cisplatin-induced apoptosis in the AGS gastric cancer cell line

**DOI:** 10.1186/1477-7819-11-5

**Published:** 2013-01-13

**Authors:** Qi Zheng, Ling-Yu Zhao, Ying Kong, Ke-Jun Nan, Yu Yao, Zi-Jun Liao

**Affiliations:** 1 Department of Medical Oncology, First-Affiliated Hospital, Xi’an Jiaotong University, 277 Yanta West Road, Xi’an, 710061, Shaanxi Province, China; 2 Affiliated Shaanxi Provincial Cancer Hospital, College of Medicine, Xi’an Jiaotong University, Xi’an, 710061, Shaanxi Province, China; 3 College of Medicine, Xi’an Jiaotong University, Xi’an, 710061, Shaanxi Province, China

**Keywords:** Gastric cancer, CDK-associated Cullin1, Proliferation, Cell cycle, Apoptosis

## Abstract

**Background:**

Gastric cancer is a common and highly lethal malignancy in the world, but its pathogenesis remains elusive. In this study, we focus on the biological functions of CDK-associated Cullin1 (*CAC1*), a novel gene of the cullin family, in gastric cancer, which may help us to further understand the origin of this malignancy.

**Methods:**

The AGS and MGC803 gastric cancer cell lines and the GES-1 gastric mucosa cell line were selected for study. At first, *CAC1* expressions of those cell lines were examined by quantitative real-time reverse transcription polymerase chain reaction (qRT-PCR) and western blot examinations, then *CAC1* small interfering RNA (*CAC1*-siRNA) were designed and transfected into the AGS cell line with a relatively high level of *CAC1*. Once *CAC1* was silenced, a series of biological characteristics of AGS cells such as cell proliferation, cell cycle, apoptosis, and expressions of apoptosis-related genes (*P53*, *BCL2* and *BAX*) were determined by MTT, flow cytometry, qRT-PCR and western blot, respectively.

**Results:**

*CAC1* expression of AGS or MGC803 was much higher than that of GES-1. After *CAC1* expression was effectively depressed by RNA interference in AGS cells, significant cell growth inhibition occurred. Furthermore, the proportion of cells treated with *CAC1*-siRNA increased in the G1 phase and decreased in the S phase, indicative of G1 cell cycle arrest. More importantly, the proportions of early/late apoptosis in AGS cells were enhanced with cis-diaminedichloroplatinum (cisplatin, CDDP) treatment, but to a higher extent with cisplatin plus *CAC1*-siRNA. Interestingly, *BCL2* mRNA copies showed about a 30% decrease in the cisplatin group, but dropped by around 60% in the cisplatin plus *CAC1*-siRNA group. Conversely, the *P53* mRNA expressions obtained nearly a two-fold increase in the cisplatin group, in addition to a five-fold increase in the cisplatin plus *CAC1*-siRNA group, and the *BAX* mRNA levels had almost a two- and four-fold augmentation, respectively. Meanwhile, *P53*, *BAX* and *BCL2* showed the same alteration patterns in western blot examinations.

**Conclusions:**

*CAC1* can promote cell proliferation in the AGS gastric cancer cell line. Moreover, it can prevent AGS cells from experiencing cisplatin-induced apoptosis via modulating expressions of *P53*, *BCL2* and *BAX*.

## Background

Gastric cancer is one of the most common malignant tumors and the second leading cause of cancer death in the world, responsible for a total of 989,600 new cases and 738,000 deaths annually [1]. Over past years, there has been a steady decline in the incidence and mortality risk of gastric cancer in most countries [2], due to the tremendous developments in diagnosis and treatment methods. However, gastric cancer remains a great threat to people, especially those in developing countries [1], and the survival of all affected patients, even after curative surgical resection and adjuvant therapy, is less than 40% [3].

Epidemiologic investigations have uncovered many risk factors for gastric cancer, and molecular biology research further indicates that gastric carcinogenesis comprises numerous genetic and epigenetic events, involving a cluster of oncogenes, tumor suppressor genes, cell cycle regulators, cell adhesion molecules and DNA repair genes [4]. However, the precise mechanisms underlying gastric cancer are not well defined.

CDK-associated Cullin1 is a novel gene identified in colorectal carcinoma. It embraces an open reading frame sequence which encodes a 37 kDa protein of 369 amino acids [5]. The *CAC1* protein contains a cullin domain between amino acids 137 and 250, and is therefore classified as a member of the cullin family of E3 ubiquitin ligases [5]. Histological investigations had established a possible association of *CAC1* expression with pathological features and clinical stages of colorectal carcinoma patients [5]. Moreover, *CAC1*, *in vitro*, was expressed in a cell cycle-dependent manner with high expression in the late G1 to S phase [5]. Notably, *CAC1* could promote cell cycle progression and stimulate the kinase activity of *CDK2*[5]. Nevertheless, little is known about its expression and biological characteristics in gastric cancer.

The present study was conducted to investigate the expression of *CAC1* and to explore the function that *CAC1* performs in gastric carcinoma cell lines. The AGS cell line was selected as the model for study because it expressed relatively high level of *CAC1*, then *CAC1* expression was silenced by RNA interference (RNAi), and a series of biological parameters relevant to cell proliferation, cell cycle and apoptosis were examined, correspondingly.

## Methods

### Cell culture

Human gastric cancer cell lines (AGS and MGC803) and gastric mucosa cell line (GES-1) were provided by Central Laboratory of Medical College, Xi’an Jiaotong University, China. Cells were cultured in RPMI 1640 medium (Gibco BRL, Grand Island, NY, USA) supplemented with 10% newborn calf serum (Gibco BRL, Grand Island, NY, USA), 100 kU/L penicillin, 0.1 g/L streptomycin, 0.3 g/L L-glutamine and 0.85 g/L NaHCO_3_ at 37°C in a humidified atmosphere containing 5% CO_2_.

### siRNA transfection

*CAC1*-siRNA (sense-GGA UGG UGC CAU AGA UCA ATT 3^′^, antisense-5^′^UUG AUC UAU GGC ACC AUC CGG3^′^), *CAC1* negative control siRNA (NC-siRNA, sense-5^′^UUC UCC GAA CGU GUC ACG UTT 3^′^, antisense-5^′^ACG UGA CAC GUU CGG AGA ATT 3^′^) were chemically synthesized by Shanghai GenePharma Corporation (SGC, Shanghai, China). All siRNAs were mixed into Lipofectamine2000 (Invitrogen, Carlsbad, California, USA) and transfected according to the siRNA Transfection Protocol. The efficiency of *CAC1* knockdown was evaluated with qRT-PCR and western blot tests.

### MTT assay

The MTT (3-[4,5-dimethylthiazol-2-yl]-2,5-diphenyltetrazolium bromide thiazolyl blue indicator dye) chemosensitivity assay was applied to determine the proliferation rate of AGS gastric cells. Cells were seeded at a concentration of 5 × 10^3^ cells per well in 96-well plates. All experiments were conducted in triplicate. Cells were incubated for 24 hours and were divided into five groups with five different treatments (null, NC-siRNA (60 nmol/L(nM)), siRNA (30nM), siRNA (60nM), siRNA (90nM)) for 0, 24, 48, 72 and 96 hours, correspondingly. Twenty microliters of 5 mg/ml MTT (Sigma Chemical Co, St. Louis, MO, USA) in phosphate buffered saline (PBS) were added per well and cells were left inside the incubator for another 4 h at 37°C, followed by the addition of 150 μl DMSO. Absorbance of the colored solution was measured by a fully automated multi-detection microplate reader (POLARstar OPTIMA, BMG Labtechnologies, Offenburg, Germany) at 490 nm.

#### Flow cytometric analysis

Cells (1 × 10^5^ cells/well) were harvested in 6-well plates for 24 hours, and were treated with different agents (null, NC-siRNA, siRNA (60nM)) for 48 hours. Then they were collected to be washed with 0.01 mol/L cold (4°C) PBS by spinning at 800 rpm, 4°C for 8 minutes, and then fixed in 4°C, 75% ethanol for a night. Fixed cells were centrifuged (as above) and washed again with PBS. Then cells were treated with 100 μl of DNase-free, RNaseA (10 mg/ml) and incubated at 37°C for 10 minutes. Finally, cells were stained with 100 μl of 100 μg/ml propidium iodide (light sensitive) and incubated at room temperature for 30 minutes. Cells from every sample were placed in Falcon tubes and read on a FAC sorter (Becton Dickinson, Franklin Lakes, NJ, USA). The cell cycle profiles were interpreted with B-D FAC Sort Cell Quest software.

#### Apoptosis analysis

Cells (1 × 10^5^ cells/well) were incubated in 6-well plates. After 24 hours, they were addressed with different agents (null, cisplatin (10 μM), cisplatin (10 μM) + NC-siRNA (60nM), cisplatin (10 μM) + siRNA (60nM)) in serum-free medium for 24 hours, then collected cells were stained with Annexin V/PI using Vybrant apoptosis assay kit No. 2 (Molecular Probes, Eugene, Oregon, USA) and analyzed by flow cytometry.

### Quantitative real-time reverse transcription PCR

#### Expression of CAC1 in gastric cancer cell lines

Total RNA was isolated from various cell lines (AGS, MGC803 and GES-1) using the acid guanidinium-phenol-chloroform method (Trizol, Invitrogen, Carlsbad, USA), and cDNAs were synthesized using the PrimeScriptTM 1st Strand cDNA Synthesis Kit (Invitrogen, Carlsbad, USA). Primer sequences used for amplification were designed by TaKaRa (Takara Bio Inc., Shiga, Japan) and listed as follows: forward primer 5'-GCA GCA TAT TCA GAA AGT TCA GA-3'; reverse primer 5'-CAT TTA CAG CCT AAT GCC TTT ACT-3'. *β-Actin* forward primer 5'- TGG CAC CCA GCA CAA TGA A -3'; reverse primer 5'- CTA AGT CAT AGT CCG CCT AGA AGC A -3'. Primers were used in regular PCR reactions with cDNA from cells so as to evaluate their proper design and synthesis. Subsequently, the PCR products were sequenced and their similarity to the desired nucleotide sequences confirmed. The relative amount of mRNA was determined using the SYBR GREEN PCR Master Mix (AB, Applied Biosystems, Foster City, California, USA) with gene-specific primers for *CAC1* or *β-Actin*. All steps were carried out according to the manufacturer’s protocol. Real-time PCR reactions were carried out on an ABI 7500 thermal cycler (Applied Biosystems, Foster City, California, USA) with the BioRad iQ5 and MyiQTM Real-time PCR Detection System (BioRad Laboratories, Hercules, California, USA). Three independent PCR tests were performed from each RT sample. The expression of *CAC1* mRNA in each sample was normalized against *β-actin* and the expression level was calculated using the ΔΔCT (delta delta threshold cycle) method.

### Expression of apoptosis-related genes in AGS cell line

RNA isolation and cDNA synthesis were performed as noted. A series of primers were designed and synthesized by TaKaRa including *P53* (forward-5^′^-CCA CCA TCC ACT ACA ACT ACA T-3^′^, reverse-5^′^-AGG ACA GGC ACA AAC ACG-3^′^), *BCL2* (forward-5^′^-CAA ATG CTG GAC TGA AAA ATT GTA-3^′^, reverse-5^′^-TAT TTT CTA AGG ACG GCA TGA TCT-3^′^), *BAX* (forward-5^′^-GAC ACC TGA GCT GAC CTT GG-3^′^;reverse-5^′^-AGG AAG TCC AGT GTC CAG C-3^′^) and *β-Actin* (forward 5′-TGG CAC CCA GCA CAA TGA A-3^′^; reverse 5^′^- CTA AGT CAT AGT CCG CCT AGA AGC A −3^′^) genes. As indicated above real-time PCR was performed three times. The PCR products were detected by measuring the emitting fluorescence at the end of each reaction cycle. The threshold cycle corresponds to the number of cycles required to detect a fluorescence signal above the baseline. The *β-Actin* gene served as the internal control of the reaction.

The mRNA expression levels were calculated using the 2^-ΔΔCT^ method and expressed in relative quantification units. In detail, the threshold cycles of the housekeeping gene *β-Actin* and the target genes *BCL2*, *BAX* and *P53* were determined in each sample and for each time period. CT values were normalized (ΔCT) by subtracting the expression levels of the reference gene *β-Actin* from the corresponding expression levels of the *BCL2*, *BAX* and *P53* in each sample. The normalized gene expression in the treated AGS cells was analyzed relative to their matching untreated cells, which acted as calibrator samples (ΔΔCT).

### Western blot examinations

Fifty micrograms of total protein from the AGS cell line was separated on a 10% Trisglycine SDS polyacrylamide gel and transferred to a nitrocellulose membrane (BioRad, Hercules, California, USA). The blot was probed with mouse monoclonal antibodies including anti-*CAC1* (GeneTex, Irvine, California, USA, 1:1500), anti-*β-Actin* (Santa Cruz, CA, USA; 1:5000), anti-*P53* (Santa Cruz, CA, USA; 1:2000), anti-*BCL2* (Santa Cruz, CA, USA; 1:2000), and anti-*BAX* (Santa Cruz, CA, USA; 1:2000). Antibody binding was detected using the Gel Blot Imaging Systems (Syngene G:BOX, Cambridge, U.K.) according to the manufacturer’s protocol.

### Statistical analyses

All statistical analyses were conducted using SPSS 13.0 software (http://www-01.ibm.com/software/analytics/spss/). Each assay was performed at least three times. The data were expressed as mean ± standard deviation, and One-way ANOVA test (two-sided) was performed to determine the significance of differences in multiple comparisons. The results were considered to be statistically significant at *P* <0.05.

## Results

### Differential expression of *CAC1* in gastric cancer and mucosal cell lines

**Figure 1:**
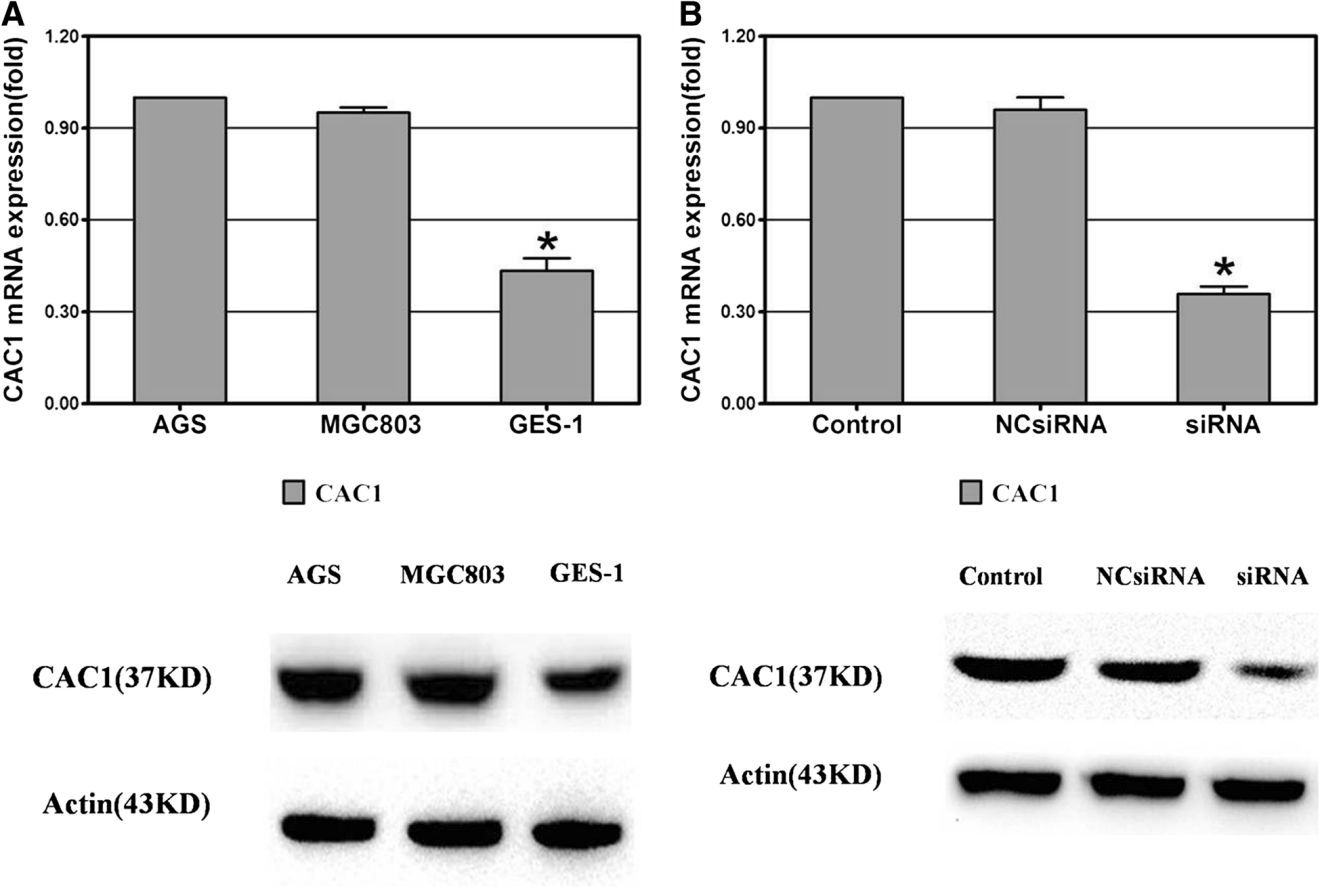
***CAC1 *expression in human gastric cancer and mucosal cell lines. (A)** Real-time reverse transcription (RT)-PCR and western blot analysis of *CAC1* expression in AGS, MGC803 and GES-1 cell lines (*represents *P *<0.05 between the GES-1 cell line and other cell lines). **(B)***CAC1 *expression was effectively depressed by 60nM *CAC1*-siRNA in the AGS cell line on the mRNA and protein level (*represents *P *<0.05 between the control group and the *CAC1*-siRNA group).

*CAC1* mRNA expression was evaluated with real-time RT-PCR in three cell lines. Obviously, *CAC1* was expressed in each of the examined cell lines (Figure 1A). However, AGS and MGC803 cell lines showed higher levels of *CAC1* mRNA than the GES-1 cell line (1.0000 ± 0.0000 and 0.9507 ± 0.0176 *versus* 0.4340 ± 0.0414, *P* <0.05). In addition, *CAC1* protein expression in western blot examinations showed the same changing trend as *CAC1* mRNA (Figure 1A).

### Endogenous expression of *CAC1* was silenced by the RNAi technique

Transient transfection of AGS cells with *CAC1*-siRNA oligos (60nM) designed against *CAC1* sequence effectively inhibited the expression of *CAC1*. *CAC1* mRNA expression was examined by real-time RT-PCR. As expected, mRNA level of *CAC1* was considerably decreased in *CAC1*-siRNA group (1.0000 ± 0.0000 *versus* 0.3583 ± 0.0244, *P* <0.05), but showed no significant difference between the control and NC-siRNA groups (1.0000 ± 0.0000 *versus* 0.9597 ± 0.0407, *P* >0.05) (Figure 1B). Meanwhile, CAC1 protein expression was also depressed after siRNA treatment in western blot examinations (Figure 1B).

### *CAC1* silencing inhibits cell proliferation in AGS cell line

**Figure 2:**
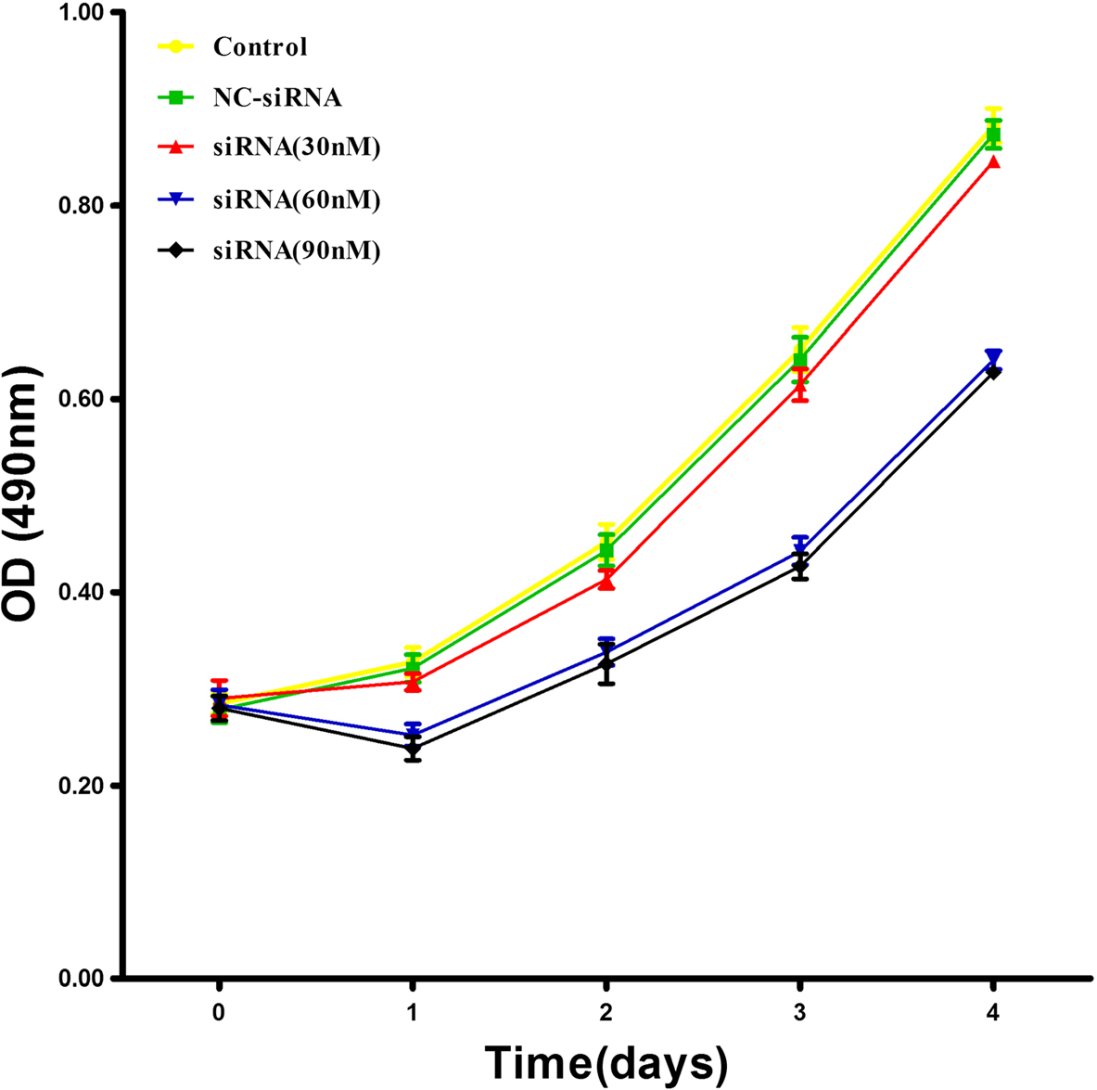
**Cell growth was inhibited following *CAC1 *silencing. **AGS cells were transiently transfected with null, NC-siRNA and three concentrations of *CAC1*-siRNA for four days, respectively. Growth rates of cells were determined by MTT assays.

In order to investigate the role *CAC1* plays in the regulation of cell proliferation, AGS cells addressed by transient transfection with *CAC1*-siRNA of three different dosages (30nM, 60nM and 90nM) were subjected to MTT assay. Within four days, *CAC1* silencing exhibited a distinct inhibitory effect on cell proliferation according to different doses of *CAC1*-siRNA (Figure 2). Optical densities (ODs) of the control group, the NC-siRNA group and the 30nM *CAC1*-siRNA group showed no significant difference with each other (*P* >0.05), but were higher than those of the 60nM and 90nM *CAC1*-siRNA groups (*P* <0.05). Interestingly, cells treated with 60nM and 90nM *CAC1*-siRNA showed almost identical ODs from the beginning to the end (*P* >0.05).

### Cell cycle analysis

**Figure 3:**
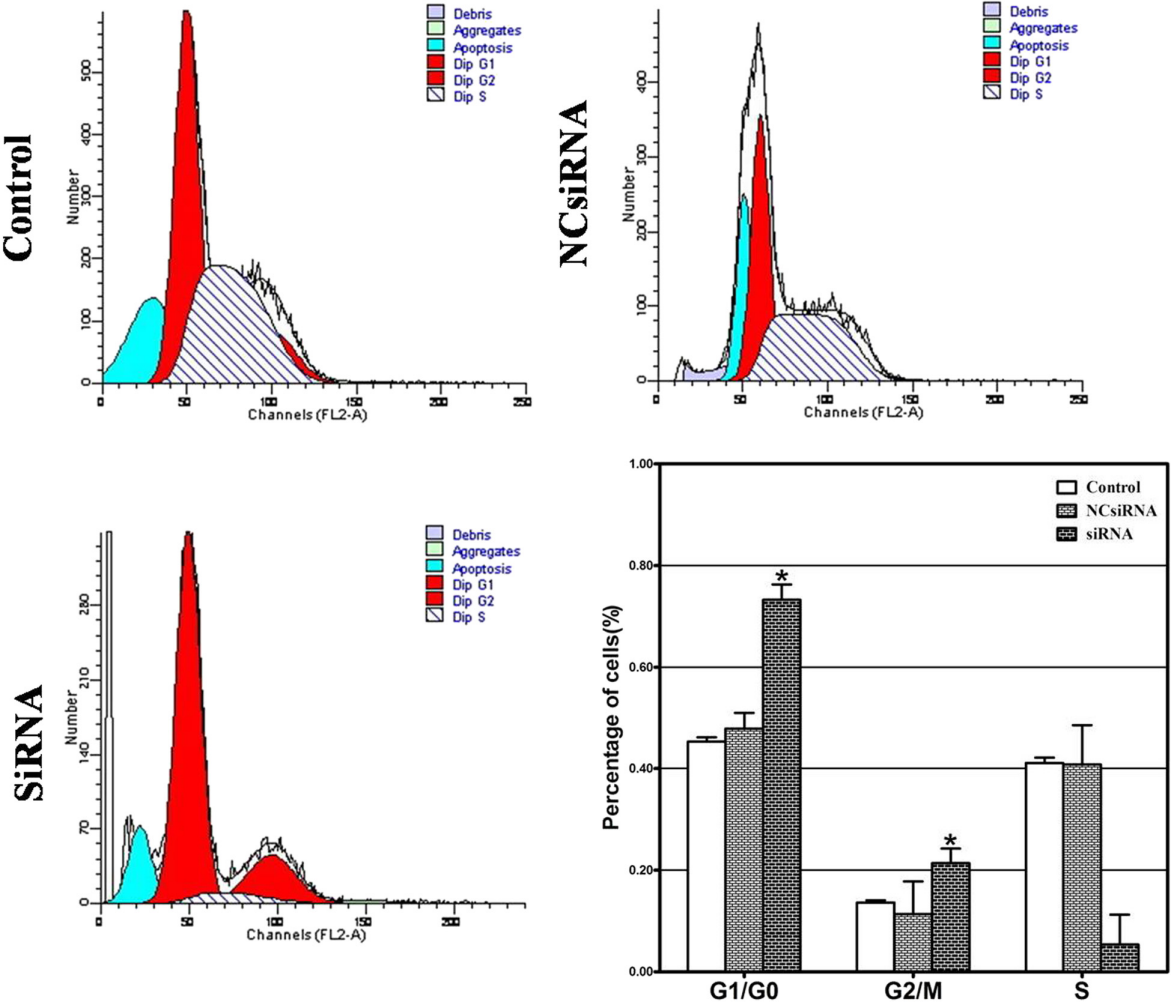
**Knockdown of *CAC1-*induced G1 cell cycle arrest in the AGS cell line. **Cell cycle analysis of AGS cells treated with or without *CAC1*-specific siRNA by flow cytometry. Proportion of cells was markedly elevated in the G1 phase while reduced in the S phase when treated with *CAC1*-siRNA (*represents *P *<0.05 between the control group and the *CAC1*-siRNA group).

Compared with the control, the proportion of cells treated with *CAC1*-siRNA increased by 28% or so in the G1/G0 phase (45.33% ± 0.82% *versus* 73.23% ± 3.04%, *P* <0.05), and decreased by approximately 36% in the S phase (41.07% ± 1.07% *versus* 5.40% ± 5.83%, *P* <0.05), with no significant change in the G2/M phase (13.61% ± 0.46% *versus* 21.37% ± 2.88%, *P* >0.05) (Figure 3). These results indicate that *CAC1* may promote cell cycle progression of AGS cells through the G1/S transition.

### Knockdown of *CAC1* enhances cisplatin-induced apoptosis

**Figure 4:**
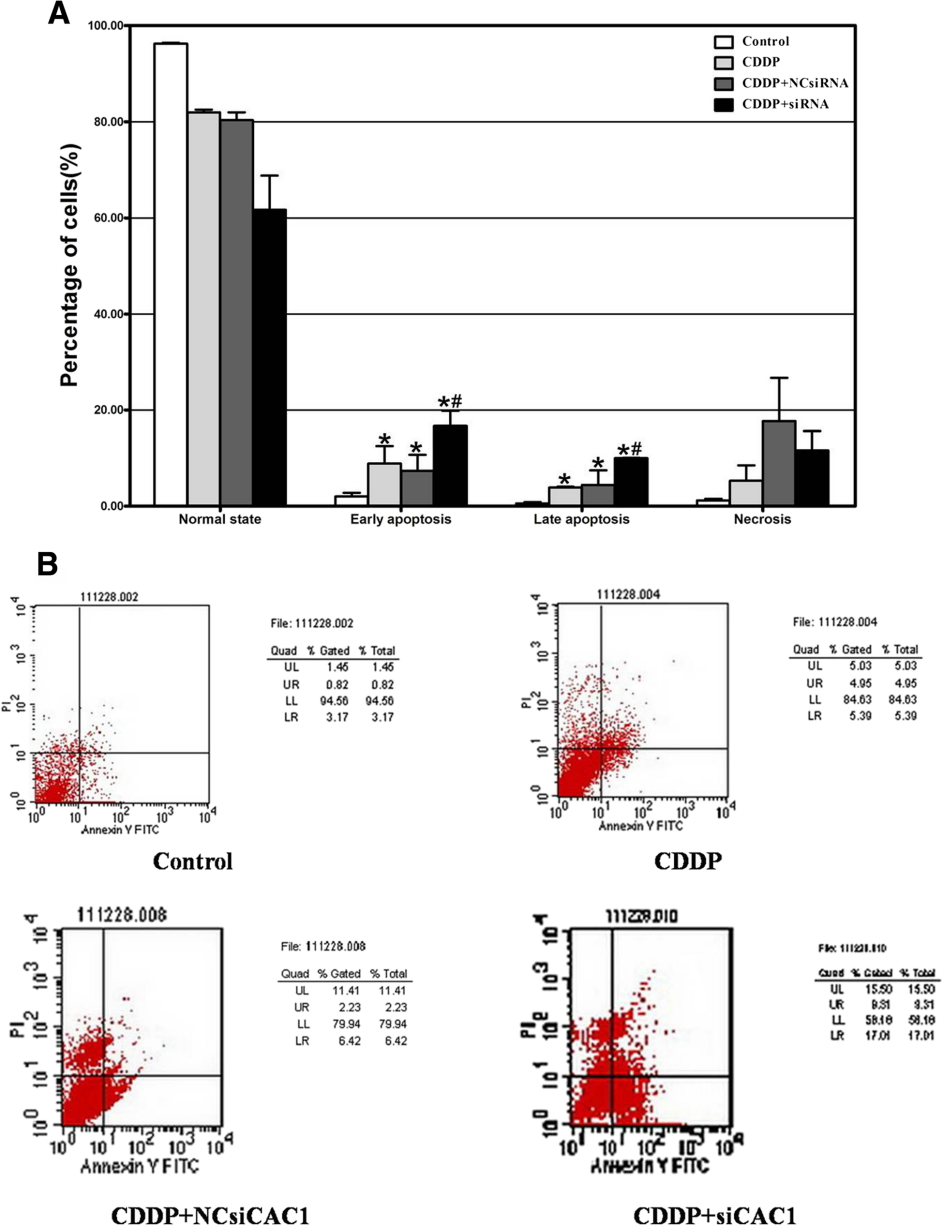
***CAC1 *silencing enhanced cisplatin-induced apoptosis in AGS cell line. (A)** The effects of cisplatin alone and in combination with NC-siRNA/*CAC1*-siRNA on early and late apoptosis of AGS cells (*represents *P *<0.05 between control group and treated groups; #represents *P *<0.05 between the cisplatin (CDDP) group and other treated groups). **(B) **Apoptosis patterns of AGS cells were analyzed by flow cytometry.

The AGS cells were divided into four experimental groups: the control group, the cisplatin-treated (10 μM) group, the cisplatin plus NCsiRNA group, and the cisplatin plus *CAC1*-siRNA (60nM) group. Compared with the control group, the proportions of early/late apoptosis were enhanced with cisplatin treatment, but to a higher extent with cisplatin plus *CAC1*-siRNA (2.01% ± 0.78% *versus* 8.87% ± 3.65% *versus* 16.69% ± 3.15%/0.57% ± 0.25% *versus* 3.89% ± 0.15% *versus* 10.01% ± 0.09%, *P* <0.05) (Figure 4A and 4B), which signified that the knockdown of *CAC1* dramatically accelerated cisplatin-induced apoptosis.

### Expression profiles of apoptosis-related genes

**Figure 5:**
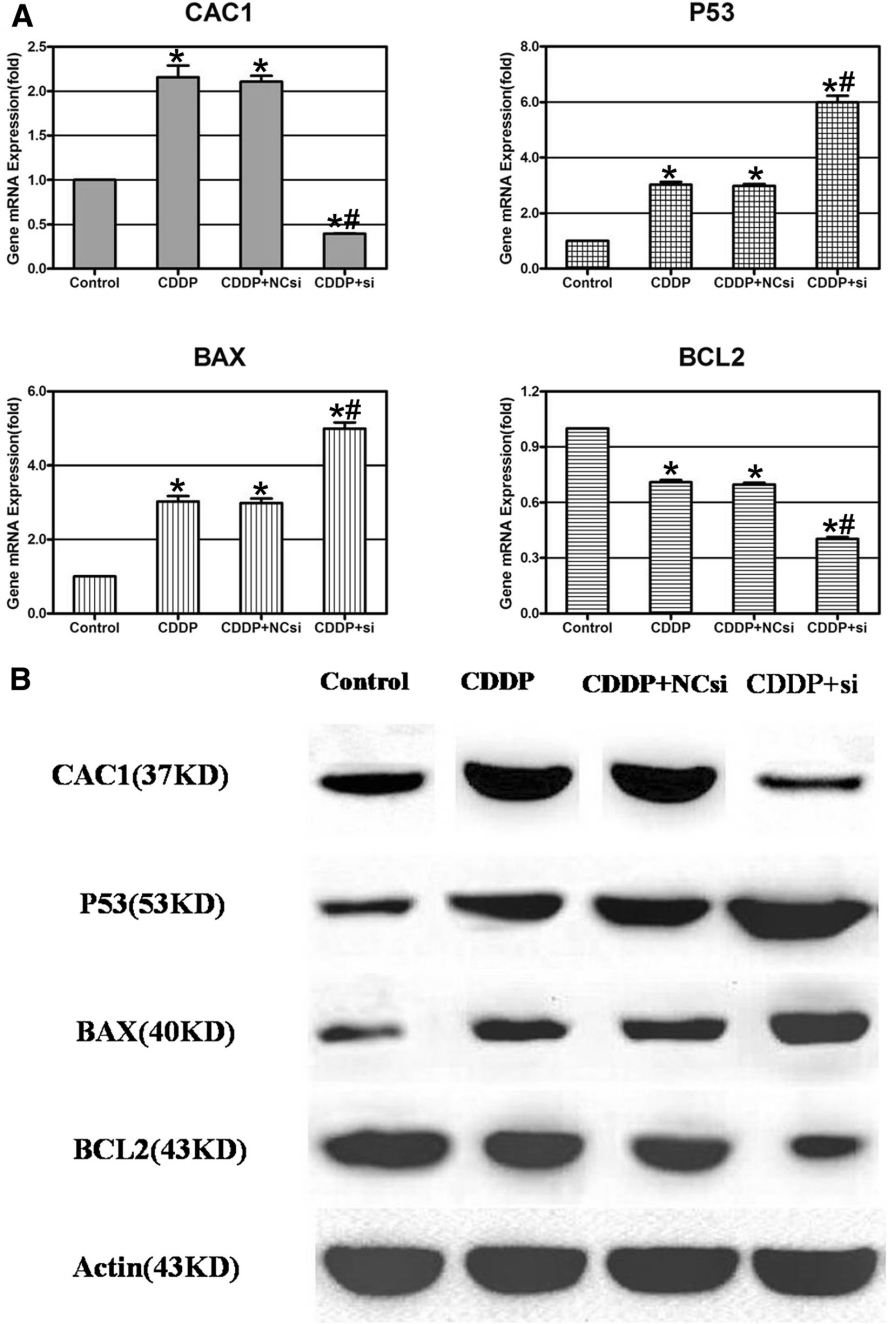
**Expression profiles of apoptosis-related genes in response to cisplatin alone or with *CAC1*-siRNA treatments. (A) **Real-time reverse transcription (RT)-PCR analysis of *CAC1*, *P53*, *BAX *and *BCL2 *mRNA expression in AGS cells (*represents *P *<0.05 between *CAC1*-siRNA group and treated groups; #represents *P *<0.05 between the cisplatin (CDDP) group and other treated groups). (**B**) Western blotting analysis of *CAC1*, *P53*, *BCL2 *and *BAX *expression in AGS cells.

The mRNA levels of *CAC1*, *BCL2*, *BAX* and *P53* were examined by qRT-PCR. Incubation of the AGS cells with 10 μM cisplatin and/or 60nM *CAC1*-siRNA for 24 h did produce interesting mRNA profiles (Figure 5A). Under cisplatin treatment, *CAC1* mRNA expression greatly increased, but dropped to a low level when *CAC1*-siRNA was added simultaneously (1.0000 ± 0.0000 *versus* 2.1578 ± 0.2222 *versus* 0.3967 ± 0.0078, *P* <0.05). Compared with the control group, *BCL2* mRNA copies showed a 30% decrease in the cisplatin group, but dropped by around 60% in the cisplatin plus *CAC1*-siRNA group (1.0000 ± 0.0000 *versus* 0.7090 ± 0.0210 *versus* 0.4030 ± 0.0171, *P* <0.05). Conversely, *P53* and *BAX* transcript levels of treated groups were vastly enhanced. Compared with the control group, the *P53* expression obtained nearly a two-fold increase in the cisplatin alone group, in addition to a five-fold increase in the cisplatin plus *CAC1*-siRNA group (1.0000 ± 0.0000 *versus* 3.0187 ± 0.1738 *versus* 5.9957 ± 0.3926, *P* <0.05); the *BAX* mRNA levels had almost a two- and four-fold augmentation (1.0000 ± 0.0000 *versus* 3.0237 ± 0.2581 *versus* 4.9897 ± 0.2923, *P* <0.05), respectively.

Then western blot tests were conducted to detect the protein levels of those genes after *CAC1* knockdown. In parallel with the aforementioned mRNA changes, *BAX* and *P53* were markedly upregulated whereas *BCL2* was downregulated on the protein level (Figure 5B).

## Discussion

The ubiquitin-proteasome system plays a pivotal role in maintaining the balance between normal growth and uncontrolled proliferation by controlling the abundance of a large variety of cellular proteins [6]. The cullin family of ubiquitin ligases, traditionally composed of *CUL1, 2, 3, 4A, 4B, 5* and *7*, represents the largest class of RING-type E3 ligases (CRLs) [6]. Without intrinsic catalytic activity, cullins serve as scaffolds that facilitate the assembly of multimeric E3 ligase complexes and transfer ubiquitin from the E2-conjugating enzyme to the substrate. Cullin-mediated substrate degradation dictates a wide range of cellular processes such as proliferation, differentiation, and apoptosis. Once the cell regulatory mechanisms of cullin encounter malfunctions or perturbations, accumulation of oncoproteins or excessive degradation of tumor suppressors will inevitably occur, which may provoke cells into malignant transformation and tumorigenesis [6]. In particular, *cullin1*, the most characterized member of the cullin family, was proven to be closely associated with gastric carcinogenesis, and its overexpression predicts poor prognosis of patients with gastric carcinoma [6,7].

The expression profiles of cancer-related genes in part reflect their biological features in the pathogenesis of cancer. Being a novel member of the cullin family, *CAC1* expression patterns have been extensively investigated in a previous study [5]. Using cDNA library analysis, *CAC1* expression was found in normal stomach, small intestine, colon, liver, lung, kidney, muscle, heart, mammary gland, uterus, brain, spleen, lymph node, with high levels in the colon and mammary gland [5]. Western blot tests additionally detected *CAC1* protein in a host of normal and cancer cell lines [5]. With regard to our study, the AGS and MGC803 gastric cancer cell lines had higher *CAC1* expression than the GES-1 gastric mucosal cell line, which indicate that *CAC1* may play an active role in gastric carcinogenesis.

Gene silencing by RNA interference is a powerful method for analyzing gene function [8]. Here, we successfully transfected NCsiRNA and three concentrations of *CAC1*-siRNA into AGS cells. MTT analyses showed that the proliferation potential of AGS cells was potently inhibited by 60nM and 90nM *CAC1*-siRNA to the same degree, but failed to be influenced by 30nM *CAC1*-siRNA or NC-siRNA. It was apparent that *CAC1* could positively affect cell proliferation in gastric cancer, which was in accord with previous studies on the HeLa cell line [5]. In MTT examinations, HeLa cells expressing Flag-*CAC1* underwent higher proliferation ability than the mock transfected control cells, and cells treated with *CAC1*-siRNA showed significantly inhibited growth rate [5]. What’s more, real-time RT-PCR and western blot analyses confirmed that the expression of *CAC1* was efficaciously blocked by 60nM *CAC1*-siRNA. Taken together, 60nM was determined to be the most suitable experimental dosage for RNAi in subsequent examinations.

As a general rule, normal cell proliferation depends on orderly and efficient cell cycle process that ensures the duplication and transmission of genetic information from one cell generation to the next. In other words, deregulation of cell proliferation always lies in an abnormal cell cycle. Under *CAC1*-silencing conditions, flow cytometry in our study revealed elevated proportion of cells in the G1/G0 phase and a reduced rate of cells in the S phase. In essence, *CAC1* knockdown induced G1 cell cycle arrest in the AGS cells, which was consistent with the results from the HeLa cell line [5]. *CAC1* was also capable of binding to *CDK2* and stimulating its kinase activity at the G1/S phase transition without greatly changing the expressions of *cyclinA*, *cyclinE*, *cyclinD1*, *CDK2*, *RB*, *PTEN*, and so on [5]. It is supposed that *CAC1* depression attenuates the activity of *CDK2* and interrupts the G1-S transition.

Apoptosis is one of the basic biological phenomena characterized by a series of transformations in cell morphology [9]. Furthermore, apoptosis in concert with cell proliferation forms a crucial balancing mechanism that manages a large number of physiological procedures such as tissue homeostasis and normal development [9]. Under pathological circumstances, aberrant apoptosis usually contributes much to the initiation and progression of cancer [10-12] and even influence the sensitivity of cancer cells to therapeutic interventions [13].

The unequivocal activity of *CAC1* in cell cycle regulation raised the possibility that it is, more or less, involved in the process of apoptosis. In the current study, the proportion of early/late apoptotic cells increased with cisplatin treatment, but increased even more so when *CAC1* expression was concurrently inhibited by RNAi. That is to say, apoptotic indices of the cisplatin plus *CAC1*-siRNA group obtained a significant increase in comparison with those of the former three groups with intact *CAC1*. Cumulative data imply that *CAC1* may weaken the anti-cancer effect of cisplatin by counteracting cisplatin-induced apoptosis.

Regulation of apoptosis, however, relies on a network of anti- and proapoptotic molecules such as *BCL2* family [14]. *BCL2* (B cell CLL/lymphoma 2) is a proto-oncogene located in the chromosomal region 18q21.3, which codes for an antiapoptotic 26-kDa protein containing four BH domains (BH1 ~ BH4)) [15]. It can hinder the release of cytochrome c from the mitochondria, thus abrogating the activation of caspases and finally inhibiting apoptosis [16]. According to early studies, the overexpression of *BCL2* drives cells toward malignant transformation [17] and predicts the prognosis in many malignancies [18-22]. Particularly in gastric cancer, frequent expression of the *BCL2* gene always occurred in malignant tissues [23-25]. High expression levels of the *BCL2* gene, though correlated with less aggression of stomach cancer [26], had a lot to do with drug resistance of the cancer cells [27].

*BAX* (*BCL2* associated X protein) gene is located in the human chromosomal region 19q13.3-q13.4 [28]. Its 21-kDa encoding protein, in particular, serves as a proapoptotic member of the *BCL2* family. *BAX* protein consists of three BH domains (BH1, BH2, and BH3) and shares a lot of homology with the *BCL2* protein. Indeed, *BAX* protein acts as a suppressor of *BCL2* to accelerate apoptotic cell death, by forming *BAX*/*BCL2* complexes or by competing with other *BCL2* targets [29]. Overexpression of the *BAX* gene had a negative effect on cell growth in human gastric cancer, owing to the induction of apoptosis and to the enhancement of cell chemosensitivity [30]. On the contrary, suppression of *BAX* gene expression induced tumorigenesis in gastric epithelia [23].

Cisplatin is a kind of chemotherapeutic agent widely used in solid malignancies including gastric cancer. It is generally accepted that its primary cytotoxic effect is DNA damage and subsequent induction of apoptosis [31], so variances of apoptosis-associated genes in cisplatin treated cells penetratingly mirror the mechanisms underlying cisplatin-induced apoptosis. As for our study, *CAC1* expression was upregulated by cisplatin treatment, but was markedly downregulated by siRNA treatment despite previous cisplatin. Furthermore, underlying the increase of cisplatin-induced apoptosis that follows *CAC1* silencing are concomitant gene expression alterations including the upregulation of *P53* and *BAX* as well as the downregulation of *BCL2.* These effects suggest that *CAC1* strengthens cisplatin-induced apoptosis by modulating the expression of *BCL2*, *BAX* and *P53*.

As mentioned previously, *BCL2* is seemingly situated at the convergence of a couple of apoptotic pathways, and the ratio of *BCL2* to *BAX* protein appears to be the final determinant of whether a cell enters the execution phase [31]. In fact, it is the *BCL2*/*BAX* ratio that governs the sensitivity of cells to apoptotic stimuli [32,33]. In the process of cisplatin-induced apoptosis, *CAC1* might protect AGS cells from apoptosis by altering *BCL2*/*BAX* ratio, for *CAC1* silencing brought out a pronounced increase of *BAX* and a decrease of *BCL2*, which was conducive to the occurrence of cell apoptosis.

Interestingly, the *P53* gene in the AGS cells was upregulated with the cisplatin treatment, especially with synchronous suppression of *CAC1*. It is well known that *P53* plays an important role in the management of cell cycle and apoptosis. DNA damage resulting from cisplatin may stimulate expression of the *P53* protein that results in both expression of downstream *P21* protein and G1 cell cycle arrest [34]. If confronted with irreparable DNA damage, the *P53* protein triggers programmed cell death [31]. During cell apoptosis, *P53* activates *BAX* via transcriptional [35] or transcription-independent [36] mechanisms, and represses transcription of *BCL2*[14]. Furthermore, *P53* can nontranscriptionally induce apoptosis [37]. Therefore, *CAC1* inhibition in the AGS cells can bring about excessive *P53* accumulation, *BAX* accumulation, and *BCL2* reduction, which ultimately potentiated apoptosis.

The mechanism by which *CAC1* functions is not fully clarified. *CAC1* was able to reinforce the activity of *CDK2*[5], and *CAC1* silencing probably impaired the CDK2 activity. An early study argued that CDK2 inhibition could lead to *ATM*- and *ATR*-dependent *P53* phosphorylation at serine 15, and thereby cause a significant increase of *P53* and *P21* protein [38]. With the potential to activate *CDK2*, *CAC1* is inclined to interfere with the *P53P21* pathway, and thus help AGS cells to resist G1 arrest and apoptosis. On the other hand, *CAC1* can serve as a corepressor of *RARα* to negatively regulate retinoid acid-induced cellular differentiation and CoRNR box is confirmed to be a major functional region of *CAC1*[39]. So it seems that CoRNR box has the potential to regulate cell proliferation and apoptosis, which deserves to be further investigated in other studies.

## Conclusion

In summary, *CAC1* exerts multiple biological effects in the AGS gastric carcinoma cell line. Through promoting proliferation and by countering cisplatin-induced apoptosis, *CAC1* has been deeply implicated in the pathogenesis and development of gastric cancer. More explorations on specific details of the molecular mechanisms are warranted in the future.

## Abbreviations

CAC1: CDK-associated Cullin1; CAC1-siRNA: CAC1 small interfering RNA; cisplatin or CDDP: cis-diaminedichloroplatinum; MTT: 3-[4,5-dimethylthiazol-2-yl]-2,5-diphenyltetrazolium bromide thiazolyl blue indicator dye; OD: optical density; PBS: phosphate buffered saline; qRT-PCR: quantitative real-time reverse transcription polymerase chain reaction; ΔΔCT: delta delta threshold cycle.

## Competing interests

All authors declare that they have no competing interests.

## Authors’ contributions

QZ, L-YZ, and K-JN designed the research. QZ, L-YZ, YK, YY, and Z-JL performed the research. QZ and L-YZ analyzed the data. QZ wrote the paper. All authors read and approved the final manuscript.
